# Brain metastases from esophageal cancer: A case report

**DOI:** 10.1097/MD.0000000000020223

**Published:** 2020-06-12

**Authors:** Shuai Qie, Yuge Ran, Huibin Yang, Guimin Cui, Miaoling Liu, Xiaojing Sun, Yuan Tian, Wenfan Sun, Nan Li, Chan Liu

**Affiliations:** Department of Radiation Oncology, Affiliated Hospital of Hebei University, Baoding, Hebei, China.

**Keywords:** brain metastases, case report, esophageal cancer, temozolomide

## Abstract

**Introduction:**

At present, there is no uniform consensus on the treatment of brain metastases from esophageal cancer. The studies on the treatment of brain metastases from esophageal cancer by radiotherapy combined with temozolomide (TMZ) are even rarer.

**Patient concerns:**

A 69-year-old woman was admitted to our hospital for brain metastases from esophageal cancer after thoracic irradiation.

**Diagnoses:**

Magnetic resonance imaging of the head showed a round, heterogeneous metastatic tumor in the left parietal lobe. Brain magnetic resonance imaging showed edema around brain metastases

Interventions: After radiotherapy plus TMZ in this patient's head, the brain metastatic tumor was significantly decreased.

**Outcomes:**

At the end of radiotherapy, and 1 and 2 months after the end of radiotherapy, the metastatic tumor continued to shrink, and no obvious side effects were observed.

**Lessons:**

This study suggests that radiotherapy plus TMZ might be a feasible option for brain metastases from esophageal cancer.

## 1 Introduction

Esophageal cancer is a primary malignant tumor of the esophagus, and squamous-cell carcinoma is the most common. Clinically, progressive dysphagia is the typical symptom. According to the 2017 cancer statistics, the number of newly diagnosed esophageal cancer is 16,940 and the death toll from esophageal cancer is 15,690.^[1]^ The exact etiology of esophageal cancer is still unclear. The occurrence of esophageal cancer is related to the living conditions, dietary habits, the presence of strong carcinogens, the lack of some anticancer factors, and genetic susceptibility. The main progression of esophageal cancer are direct diffusion, lymph node metastasis, and hematogenous metastasis. Brain metastasis of esophageal carcinoma is rare, with an incidence of 0.4% to 5.1%, and the prognosis is also poor.^[2]^

In this case report, we successfully treated a patient with brain metastasis from esophageal cancer by brain radiotherapy and concurrent temozolomide (TMZ).

## 2 Case history

**Figure 1:**
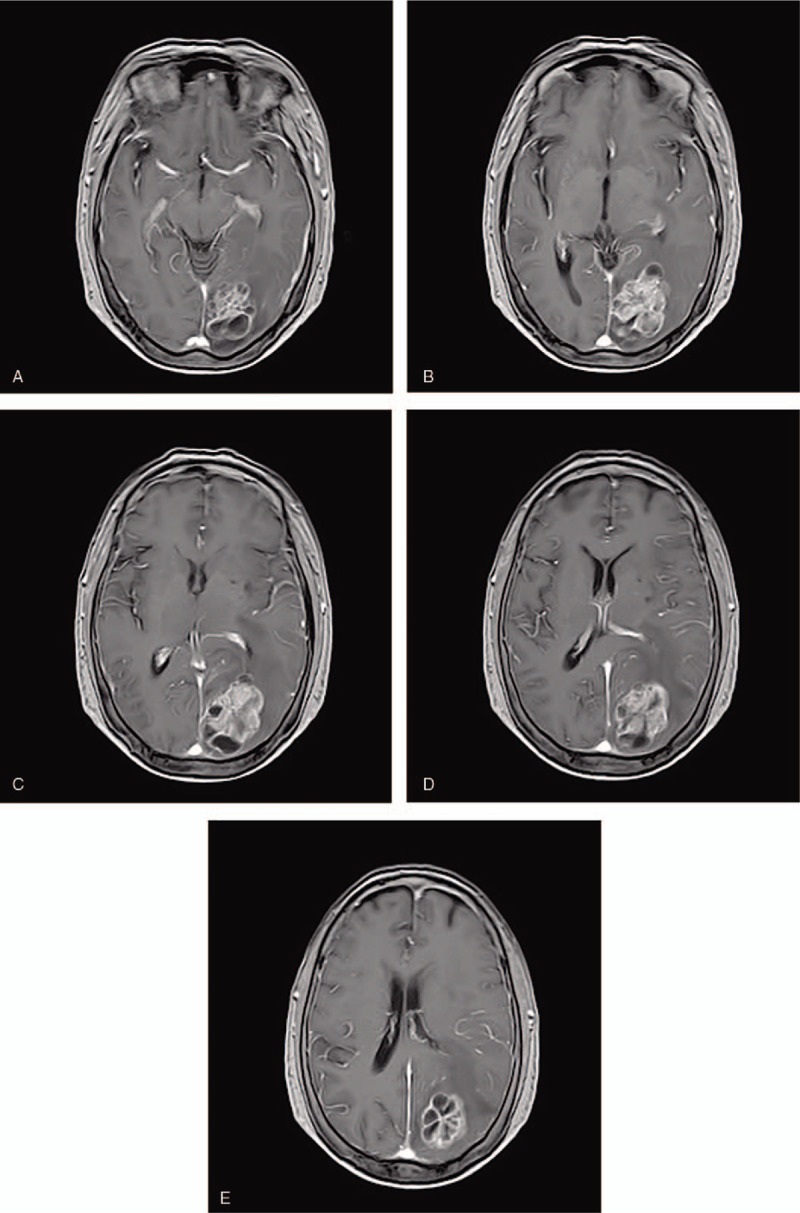
(A–E) Magnetic resonance imaging of the patient with diagnosis of left occipital metastases.

**Figure 2:**
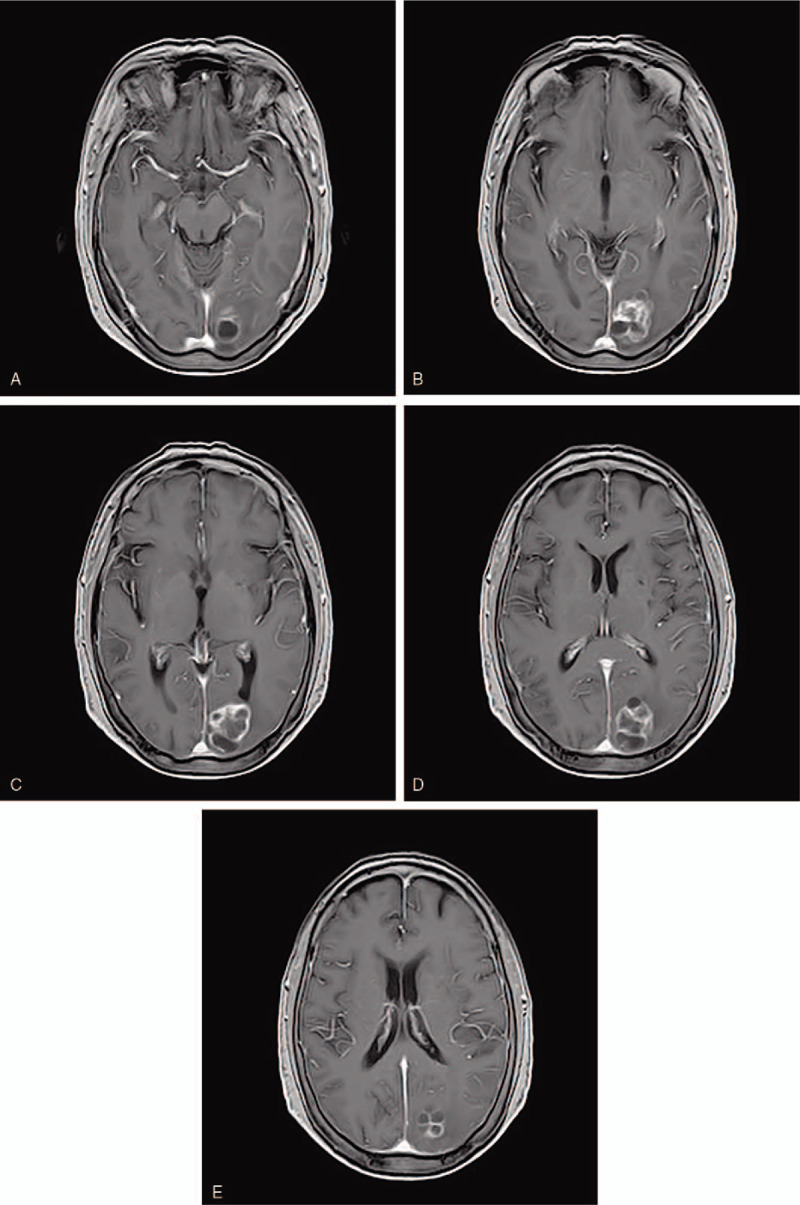
(A–E) Magnetic resonance imaging of left occipital metastasis at the end of radiotherapy.

**Figure 3:**
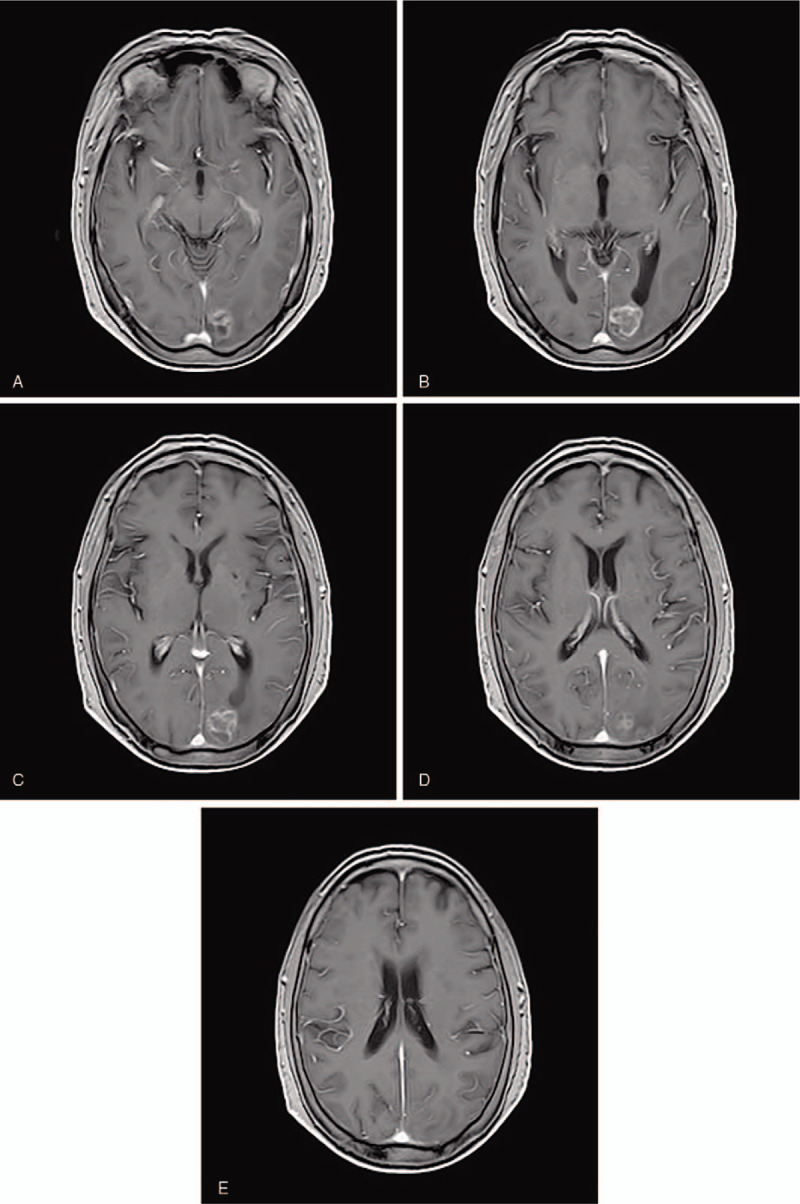
(A–E) Magnetic resonance imaging of left occipital metastasis 1 mo after radiotherapy.

**Figure 4:**
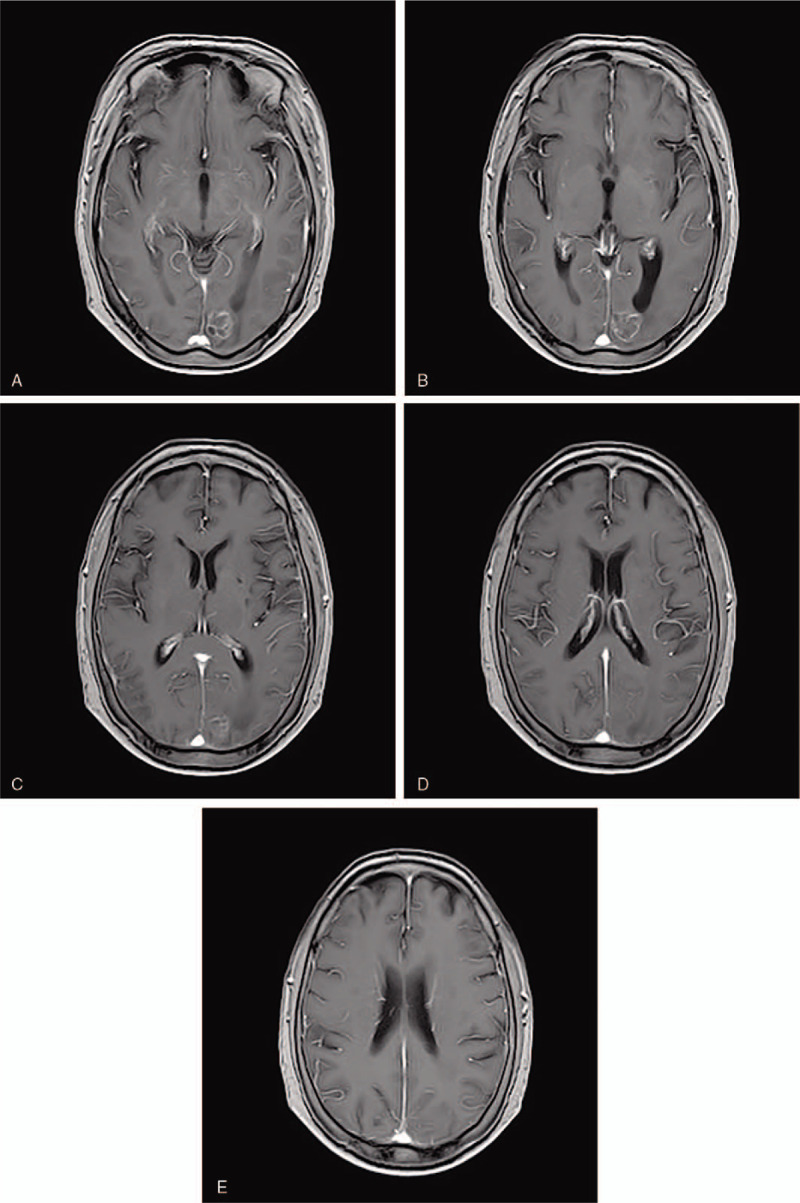
(A–E) Magnetic resonance imaging of left occipital metastasis 2 mo after radiotherapy.

A 69-year-old female patient was admitted to the esophagoscope in December 2017 due to eating difficulties, and a tumor was observed 24 to 32 cm from the incisor. The pathological diagnosis was squamous-cell carcinoma. immunohistochemical markers:P40(+),CK5/6(+),CD56(+),Syn(-),NSE(-),Ki67(+70%). The Karnofsky performance status (KPS) score at diagnosis was 80. She underwent radiotherapy for esophageal cancer in her chest from December 2017 to January 2018. During the follow-up period, headache, dizziness, and instability in walking occurred in October 2018. Left parietal lobe metastasis was observed on the head magnetic resonance imaging (MRI) (Fig. 1A–E). Reexamination of chest enhanced computed tomography scan (CT) and esophagography showed no tumor recurrence. The patient received brain radiotherapy for head metastatic tumor from October 2018 to November 2018. The prescription dose of was: GTV60 Gy/20 fractions. TMZ was concurrently administrated with brain radiotherapy. The dose of TMZ was 75 mg/m^2^ daily concurrent with radiotherapy. The patient took temozolomide orally only during radiotherapy and she did not continue to take it orally after radiotherapy. At the end of brain radiotherapy (Fig. 2 A–E), 1 month (Fig. 3 A–E) and 2 months (Fig. 4 A-E) later after the end of radiotherapy, the reexamination of the head MRI showed that the left parietal lobe metastasis continued to shrink. According to standard RECIST, the efficacy was evaluated as partial response. During chemoradiotherapy, there was no side effect of digestive tract, and the toxicity of bone marrow was grade I. Headache, dizziness, and instability in walking were significantly relieved. Informed written consent was obtained from the patient for publication of this case report and accompanying images. This patient has been followed up since the end of treatment, and no recurrence of brain metastases has been observed at the time of this article.

## 3 Discussion

Esophageal cancer is a malignant tumor with high mortality. The early stage of esophageal cancer lacks typical symptoms, and the symptoms and signs of distant metastasis often contribute to the diagnosis of esophageal cancer, but at the same time it suggests a poor prognosis. Twenty percent to thirty percent of patients with esophageal cancer had distant metastasis at the time of diagosis,^[16]^ and most patients with early stage also had local recurrence or distant metastasis soon after surgery. The metastatic sites of esophageal cancer are mainly lung, liver, bone, lymph nodes, abdominal cavity, etc,^[16]^ while the incidence of brain metastasis is very rare, especially after combined surgery, radiotherapy, chemotherapy, and other comprehensive treatment.

The interval between diagnosis of esophageal cancer and the occurrence of brain metastasis has been reported to be 5.6 to 12.3 months, and advanced and tumor burden of esophageal cancer patients are at high risk of brain metastasis.^[3]^ Ogawa reported on 32 cases of patients with brain metastases from esophageal cancer, 26 cases (81%) patients with brain metastases were stage III–IV.^[4]^ Weinberg showed that 27 cases of esophageal cancer, 19 (70%) cases of stage IV were brain metastases.^[5]^ Wu's^[6]^ findings confirmed that esophageal adenocarcinoma was more prone to brain metastasis compared to squamous cell carcinomas. A retrospective study from China showed that the prognosis of patients with brain metastasis from esophageal cancer was poor. For single brain metastases, surgery and good KPS might indicate a good prognosis.^[7]^

Due to the lack of relevant prospective randomized controlled clinical studies or large meta-analyses of different therapeutic effects, there is no reported guideline for treatment at present. Treatment is based on the location of the tumor, size, and the number of brain metastases, taking various factors into consideration.^[2]^ Treatment includes surgery, whole brain radiotherapy (WBRT), stereotactic radiotherapy, and chemotherapy alone or in combination. The purpose of surgery is to improve the symptoms of the nervous system, locally control tumor metastasis and improve the prognosis of patients. Surgery is the standard treatment for single brain metastases,^[17]^ especially for patients with limited lesions and better general conditions. Previous studies have suggested that resection of brain metastases can be used for diagnosis and 1 to 3 lesions or lesions with a small number but close range and primary tumor could be well controlled.^[8]^

WBRT has long been used as one of the main treatment method for brain metastasis. Compared with support therapy alone, WBRT can improve the symptoms of patients and the median survival time.^[18,19]^ In addition, WBRT can also control the emergence of new metastatic lesions, reduce neurological defects and the need for rescue treatment, and control micro-metastatic lesions that are not found in MRI. A randomized controlled trial compared the efficacy of surgery alone versus surgery combined with WBRT in patients with single brain metastases. Local recurrence was significantly reduced by radiotherapy, but there was no difference in overall survival between the 2 groups.^[9]^ There was increasing evidence that small size metastasis was a better choice for stereotactic radiotherapy than the number of metastases.^[10–12]^ All 3 studies showed that the volume of metastases was a better predictor of survival than the number of metastases.

Chemotherapy is rarely used for brain metastases. In 2 randomized controlled trials, the addition of carboplatin or TMZ to WBRT did not improve overall survival compared with radiotherapy alone. However, there wereclinical reports that TMZ can improve progression-free survival or increase radiation response.^[13–15]^

In conclusion, for the treatment of patients with brain metastasis from esophageal cancer, clinicians should consider comprehensively the stage of primary tumor, the age of patients, KPS score and the size of brain metastases and location of the metastases to choose a reasonable treatment plan. The research on brain metastasis from esophageal cancer is limited, and it is not as sufficient as that of lung cancer and breast cancer. Therefore, more clinical and basic studies are needed to improve the understanding of brain metastasis from esophageal cancer. It is believed that, with the progress of diagnostic methods, in-depth study of tumorigenesis mechanism and the development of new targeted drugs, survival of patients with brain metastasis from esophageal cancer will be further improved.

## Author contributions

**Data curation:** Huibin Yang.

**Formal analysis:** Guimin Cui.

**Investigation:** Miaoling Liu.

**Methodology:** Xiaojing Sun.

**Software:** Yuan Tian.

**Supervision:** Nan Li.

**Validation:** Wenfan Sun.

**Visualization:** Chan Liu.

**Writing – Original Draft:** Yuge Ran.

**Writing – review & editing:** Shuai Qie.
